# Clinical characteristics and prognostic factors for short-term outcomes of autoimmune glial fibrillary acidic protein astrocytopathy: a retrospective analysis of 33 patients

**DOI:** 10.3389/fimmu.2023.1136955

**Published:** 2023-06-07

**Authors:** Wanwan Zhang, Yinyin Xie, Yali Wang, Fengxia Liu, Li Wang, Yajun Lian, Hongbo Liu, Cui Wang, Nanchang Xie

**Affiliations:** ^1^ Department of Neurology, the First Affiliated Hospital of Zhengzhou University, Zhengzhou, China; ^2^ Department of Neurology, Henan Children’s Hospital, Zhengzhou, China; ^3^ Department of Clinical Laboratory, the First Affiliated Hospital of Zhengzhou University, Zhengzhou, China

**Keywords:** glial fibrillary acidic protein, autoimmune, inflammatory CNS disease, Short-term prognosis, clinical characteristics

## Abstract

**Background:**

Autoimmune glial fibrillary acidic protein astrocytopathy (GFAP-A) is a recently discovered inflammatory central nervous system (CNS) disease, whose clinical characteristics and prognostic factors for short-term outcomes have not been defined yet. We aimed to assess the symptoms, laboratory tests, imaging findings, treatment, and short-term prognosis of GFAP-A.

**Methods:**

A double-center retrospective cohort study was performed between May 2018 and July 2022. The clinical characteristics and prognostic factors for short-term outcomes were determined.

**Results:**

We enrolled 33 patients with a median age of 28 years (range: 2–68 years), 15 of whom were children (<18 years). The clinical spectrum is dominated by meningoencephalomyelitis. Besides, we also found nausea, vomiting, poor appetite, and neuropathic pain in some GFAP-A patients, which were not mentioned in previous reports. And adults were more prone to limb numbness than children. Magnetic resonance imaging revealed lesions involving the brain parenchyma, meninges, and spinal cord, exhibiting patchy, linear, punctate, and strip T2 hyperintensities. First-line immunotherapy, including corticosteroid and gamma globulin, was effective in most patients in the acute phase (P = 0.02). However, patients with overlapping AQP4 antibodies did not respond well to first-line immunotherapy and coexisting neural autoantibodies were more common in women. Additionally, the short-term prognosis was significantly better in children than in adults (P = 0.04). Positive non-neural autoantibodies and proven viral infection were independent factors associated with poor outcomes (P = 0.03, 0.02, respectively).

**Conclusion:**

We expanded the spectrum of clinical symptoms of autoimmune GFAP-A. The clinical symptoms and short-term prognosis differed between children and adults. Positive non-neural autoantibodies and proven viral infection at admission suggest a poor short-term prognosis.

## 1 Introduction

Glial fibrillary acidic protein (GFAP) is an intermediate filament protein that is mainly found in the astrocytic cytoplasm, and is involved in numerous astrocyte functions. The size of GFAP lies between that of microfilaments and microtubules (1). Tissue-based assays and cell-based assays (CBA) can be used to identify the immunoglobulin G (IgG) reactive with GFAP in the cerebrospinal fluid (CSF) or serum of patients with autoimmune GFAP astrocytopathy (GFAP-A), which is a novel inflammatory central nervous system (CNS) disease reported in 2016 (2, 3). Patients usually present with meningitis (headache and neck stiffness), encephalitis (psychiatric symptoms, seizures, tremor, or delirium), myelitis (weakness and sensory symptoms), optic neuritis (blurred vision), or a combination of the above (4). The characteristic imaging feature is perivascular radial enhancement perpendicular to the ventricles, which resolves with immunotherapy (5). Coexisting neural autoantibodies are common in autoimmune GFAP-A, which makes diagnosis difficult (6). Most patients respond well to first-line immunotherapy, including corticosteroids, intravenous immunoglobulin, and plasma exchange, alone or in combination, but some are prone to relapse or death (7).

Since autoimmune GFAP-A is a recent discovery, the complete range of clinical and imaging phenotypes is still unknown. Although several GFAP-A case series have been reported, to our knowledge, no study has identified the prognostic factors for short-term outcomes in GFAP-A. Therefore, we included 33 GFAP-A patients from two hospitals in China, and retrospectively analyzed the clinical manifestations, magnetic resonance imaging (MRI) findings, laboratory examination results, treatment, and short-term prognosis. This study aims to provide new insights and improve the clinicians’ understanding of autoimmune GFAP-A.

## 2 Methods

### 2.1 Study design and participants

In this double-center retrospective observational cohort study, we enrolled patients who presented with meningitis, encephalitis, and myelitis, and tested positive for GFAP antibodies in the CSF between May 1, 2018 and April 1, 2022 at the First Affiliated Hospital of Zhengzhou University and Henan Children’s Hospital. This study was approved by the Ethics Committee of the First Affiliated Hospital of Zhengzhou University (number 2022-KY-0053) and all patients provided their informed consent.

Demographic data, clinical manifestations, CSF examination, serological tests, imaging findings, intensive care unit admission, mechanical ventilation, treatment, and outcomes were recorded. CSF examination included white cell counts, protein level, glucose level, virus antibodies detection, and oligoclonal antibodies (IgG), which were assessed in all patients with GFAP-A. Neutrophilic granulocyte, monocyte, lymphocyte, blood sodium, non-neural autoantibodies, tumor markers, and virus antibodies detection were comprised in the serological tests. Tumor markers comprised ferritin, neuron-specific enolase, alpha-fetoprotein, carcino-embryonic antigen, tumor associated antigen 125, 19-9, 15-3, and 72-4, and non-small cell lung cancer antigen 21-1. Non-neural autoantibodies contained antinuclear, anti-endothelial cell, anti-cardiolipin, anti-neutrophil cytoplasmic, anti-double-stranded DNA, anti-RA33, rheumatoid factor, anti-PM-Scl antibody, anti-SSA, and anti-Ro52 antibodies. Virus antibodies detection in CSF and serum included Epstein-Barr virus, cytomegalovirus, coxsackie virus, measles virus, herpes simplex virus I and II, human parvovirus B-19, influenza b virus, parainfluenza virus, adenovirus, rubella virus, herpes zoster virus, and echovirus. All patients underwent the above serological tests except for one patient who did not undergo virus screenings in serum. Patients exhibiting symptoms such as fever, cough, fatigue, nausea, or vomiting that cannot be explained by other causes and who were positive for the virus antibodies (immunoglobulin M) in serum, were defined as proven viral infection. If they only showed symptoms associated with viral infection without laboratory evidence, patients were defined as suspected viral infection. First-line immunotherapy included intravenous methylprednisolone (IVMP), intravenous immunoglobulin (IVIG), or plasma exchange (PE). Second-line immunotherapy included rituximab (RIT), tacrolimus (TAC), and mycophenolate mofetil (MMF). The Modified Rankin Scale (mRS) was used to evaluate the neurological status at admission, 4 weeks after the initiation of immunotherapy, and last follow-up. A favorable outcome was defined as an mRS score of <3, while a poor outcome was defined as an mRS score of ≥3. If the patient died, the mRS score was recorded as 6. All patients were followed up by telephone or in the outpatient clinic, and the last date for follow-up was July 1, 2022. We defined patients younger than 18 years as children. Relapse was defined as hospital readmission for meningoencephalomyelitis.

### 2.2 Antibody assay

The CSF and serum samples of the patients were simultaneously obtained before treatment and sent to the Neurology Laboratory of the First Affiliated Hospital of Zhengzhou University or Zhengzhou Jinyu Clinical Laboratory Center. Both institutes used fixed CBA for confirmation, with 100% agreement among positive results.

**Figure 1:**
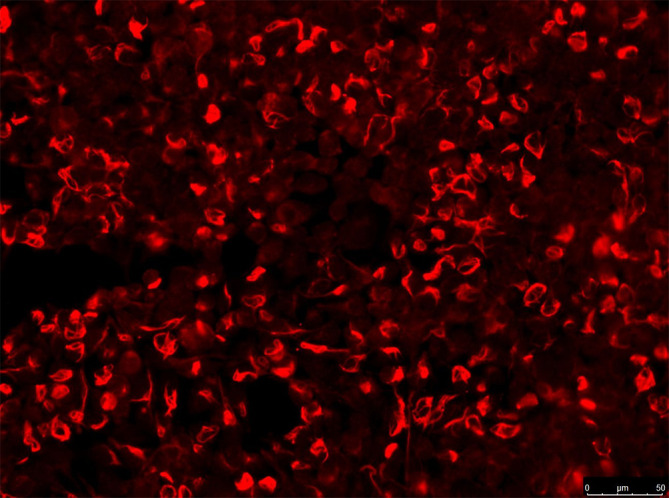
IgG in serum of patient (no.5, see **Table 1**). Those stained in red alone are GFAP antibodies. Images were obtained using a Zeiss Axiovert A1 fluorescence microscope.

The CBA method used human embryonic kidney 293 cells transfected with plasmids (pc DNA3.1) encoding GFAP homo sapiens transcript variant (NM_002055) (Shanghai Genechem Co.,Ltd) using Lipofectamine 2000. 36 hours after transfection, cells were fixed with 4% paraformaldehyde for 20 min and permeabilized with PBS containing 0.25% Triton-100 for 30 min at room temperature. Cells were incubated for 30 min at room temperature (serum diluted at 1:10, and CSF 1:1). The fluid in the wells was removed and washed 3 times with PBS afterward. AlexaFluor 546 anti-human IgG (1:500; Thermo Scientific) was used as the secondary antibody to label autoantibodies for 1 h at room temperature. Images were obtained using a Zeiss Axiovert A1 fluorescence microscope (**Figure 1**).

In addition, autoantibodies to aquaporin-4 (AQP-4), myelin oligodendrocyte glycoprotein (MOG), myelin basic protein, N-methyl-D-aspartate receptor (NMDAR), glycine receptor, glutamic acid decarboxylase 65, γ−amino butyric acid type A receptor and B receptor, α-amino-3-hydroxy-5-methyl-4-isoxazol-propionic acid receptors 1 and 2, contactin-associated protein 2, leucine-rich glioma-inactivated protein 1, Purkinje cell type 1 (Yo), antineuronal nuclear antibodies type 1 and 2, were detected with fixed CBA to examine possible coexisting autoimmunity.

### 2.3 Statistical analysis

Statistical analysis was performed using SPSS IBM 25.0 (SPSS Inc., Chicago, IL, USA). We used the fisher exact test to analyze the difference of short-term prognosis and abnormal spine cord MRIs between children and adults. The rank sum test was used for the effectiveness of first-line immunotherapy. The correlation analysis of the disease severity on admission or short-term prognosis with GFAP antibody titers and the number of symptoms was examined by Spearman’s rank correlation. Univariable binary logistic regression models were used to assess the factors affecting the outcome, and factors associated with a poor outcome (P < 0.1) were included in the multivariate binary logistic regression model. All statistical tests were two-sided, and P < 0.05 was considered statistically significant in the multivariate binary logistic regression analysis.

## 3 Results

Overall, we identified 33 patients with positive GFAP antibodies in the CSF from May 2018 to April 2022. Of these, 25 patients were from the First Affiliated Hospital of Zhengzhou University, and 8 pediatric patients were from Henan Children’s Hospital. The demographics and clinical characteristics of the patients with autoimmune GFAP-A are summarized in **Table 1**.

**Table 1:** Clinical features, auxiliary examinations, treatment strategies, and short-term prognosis in patients positive for GFAP-IgG.

Patient no. sex/age of onset	Summary clinical symptoms	MRI findings	Antibody titer		CSF White Blood Cell Count,/L; Protein, g/L; Glucose, mmol/L	Therapy	mRS at admission/4 weeks after the initiation of immunotherapy	ICU admission
			Serum antibody	CSF antibody				
1.M/2	Fever, nausea, constipation	**Brain:** T2-hyperintense lesions in bilateral frontal and left parietal **Spine:** NA	GFAP-IgG (1:100)	GFAP-IgG (1:32)	55; 273.7; 3	IVMP, acyclovir	1/0	Yes
2.F/3	blurred vision	**Brain:** T2-hyperintense lesions in bilateral frontal, parietal and temporal, corpus callosum, pons, right cerebellum, optic nerve **Spine:** lesions in C4-T8	GFAP-IgG (1:32) MOG-IgG (1:100)	GFAP-IgG (1:32) MOG-IgG (1:10)	22; 407; 2.79	IVMP, penciclovir	3/0	No
3.M/3	Fever, nausea, vomiting, lethargy, poor appetite, limb weakness	**Brain:** T2-hyperintense lesions in bilateral cerebral hemisphere, mesencephalon and cerebellum, corpus callosum, right basal ganglia, cerebellar **Spine:** normal	GFAP-IgG (1:100)	GFAP-IgG (1:32)	1;211; 3.43	IVMP, IVIG, penciclovir	5/4	Yes
4.M/7	Fever, dizziness, vomiting, abdominal pain, abdominal distension, epilepsy, consciousness disturbance, limb weakness, dysuria, constipation	**Brain:** T2-hyperintense lesions in cerebellum, bilateral basal ganglia and thalamus, left frontal and parietal **Spine:** lesions in C2-T11	GFAP-IgG (1:32)	GFAP-IgG (1:32)	138; 751.1; 3.36	IVMP, IVIG, acyclovir	5/3	No
5.M/7	vomiting, poor appetite, limb pain, fever, abdominal pain, lethargy	**Brain:** T2-hyperintense lesions in bilateral frontal, left basal ganglia and parietal **Spine:** normal	GFAP-IgG (1:100)	GFAP-IgG (1:32)	31; 381.6; 3.83	IVMP, IVIG, acyclovir	2/4	Yes
6.M/7	Dizziness, headache, fever, abdominal pain, nausea, vomiting, psychosis	**Brain:** T2-hyperintense lesions in left frontal, bilateral lateral ventricles **Spine:** lesions in T10-S2	Antibody (-)	GFAP-IgG (1:3.2)	98; 1074.0; 2.66	IVMP, IVIG, penciclovir	3/1	No
7.M/8	Fever, headache, vomiting, poor appetite	**Brain:** T2-hyperintense lesions and enhancement in meninges **Spine:** lesions and enhancement in intermittent spinal cord segments below C5	Antibody (-)	GFAP-IgG (1:100)	165; 583.4; 2.26	IVMP	2/0	No
8.F/8	Headache, nausea, limb weakness	**Brain:** T2-hyperintense lesions in bilateral frontal and lateral ventricle, left thalamus **Spine:** normal	GFAP-IgG (1:32)	GFAP-IgG (1:32)	94; 844.0; 3.29	IVMP, acyclovir	3/0	No
9.F/10	Lethargy, epilepsy, fever, limb weakness, coughing when drinking water, blurred vision	**Brain:** T2-hyperintense lesions in bilateral cerebral peduncle, parietal, and temporal, pons, medulla oblongata, left frontal, occipital, basal ganglia and thalamus. **Spine:** lesions in C3-T12	GFAP-IgG (1:32)	GFAP-IgG (1:32)	4; 273.0; 3.00	IVMP, IVIG	4/0	No
10.F/11	Fever, cough	**Brain:** normal **Spine:** T2-hyperintense lesions in T8-L1	Antibody (-)	GFAP-IgG (1:100)	156; 664.4; 2.10	IVMP	1/0	No
11.F/11	Limb weakness, lethargy, poor appetite	**Brain:** normal **Spine:** T2-hyperintense lesions in C2-6 and T9-12	GFAP-IgG (1:32) NMDAR-IgG (1:10) MOG-IgG (1:100)	GFAP-IgG (1:100) NMDAR-IgG (1:10) MOG-IgG (1:100)	18; 262.0; 2.95	IVMP, IVIG, PE, acyclovir	5/0	Yes
12.M/12	Cough, fever, poor appetite	**Brain:** T2-hyperintense lesions and enhancement in meninges **Spine:** normal	GFAP-IgG (1:32)	GFAP-IgG (1:32)	128; 1097.6; 1.00	IVMP, IVIG, acyclovir, voriconazole, amphotericin B, ceftriaxone, rifamycin	3/0	No
13.F/12	Vomiting, intermittent blurred vision, walk unsteadiness	**Brain:** normal **Spine:** T2-hyperintense lesions in medulla oblongata and C1-C5	AQP4-IgG (1:320)	GFAP-IgG (1:32) AQP4-IgG (1:100)	31; 443.0; 3.48	IVMP, IVIG, penciclovir	3/3	No
14.M/14	Fever, cough, headache, dysuria, lethargy, vomiting, limb-shaking, limb weakness,	**Brain:** T2-hyperintense lesions in right lateral ventricle, corpus callosum **Spine:** lesions in C3-7, thoracic	Antibody (-)	GFAP-IgG (1:32)	304; 4433.0; 2.31	IVMP, IVIG, penciclovir	5/0	Yes
15.F/14	Fever, headache, vomiting, limb weakness, dysuria, constipation	**Brain:** T2-hyperintense lesions in meninges, bilateral basal ganglia and thalamus **Spine:** lesions in C4-T1 and T7-L1 Enhancement in meninge and C3-7	GFAP-IgG (1:100)	GFAP-IgG (1:32)	107; 1243.9; 3.00	IVMP, acyclovir	5/0	Yes
16.F/26	Fever, headache, nausea, vomiting, dysuria, constipation, blurred vision, limb weakness, epilepsy	**Brain:** T2-hyperintense lesions in bilateral cerebellum and thalamus **Spine:** normal	GFAP-IgG (1:32) MOG-IgG (1:10)	GFAP-IgG (1:100)	38; 359.5; 2.04	IVMP, MMF, penciclovir	3/0	No
17.F/28	headache, nausea, vomiting, fever	**Brain:** T2-hyperintense lesions and enhancement in meninges **Spine:** lesions and enhancement in C2-T12	Antibody (-)	GFAP-IgG (1:32)	950; 1837.9; 1.14	IVMP, IVIG, penciclovir	3/3	No
18.F/31	Headache, dizziness, lethargy, psychosis	**Brain:** T2-hyperintense lesions in bilateral frontal and lateral ventricles Enhancement in the optic chiasm, bilateral anterior portion of the thalamus, fornix column and triventricular area **Spine:** normal	Antibody (-)	GFAP-IgG (1:1) NMDAR-IgG (1:3.2) glutamic acid decarboxylase 65-IgG (1:3.2) glycine receptor -IgG (1:1) AQP4-IgG (1:3.2)	30; 296.7; 3.55	IVMP, IVIG	4/5	No
19.M/32	Fever, headache, tics, psychosis, dysuria	**Brain:** T2-hyperintense lesions in bilateral frontal, basal ganglia, lateral ventricle, cerebellum and thalamus, left hippocamp, pons, medulla oblongata us **Spine:** normal	GFAP-IgG (1:100)	GFAP-IgG (1:3.2)	207; 2402.0; 2.51	IVMP, MMF, penciclovir	5/5	Yes
20.M/32	Dizziness, limb weakness, poor appetite	**Brain:** T2-hyperintense lesions in right frontal **PET-CT:** cervicothoracic spinal cord segmental metabolic activation	YO- IgG (1:10)	GFAP-IgG (1:32) YO- IgG (1:10)	32; 656.4; 3.53	IVMP, etoricoxib	3/4	No
21.F/35	Limb numbness, tics	**Brain:** NA **Spine:** T2-hyperintense lesions in medulla oblongata, C1-6 and T3-5	AQP4-IgG (1:100)	GFAP-IgG (1:32) AQP4-IgG (1:32)	2; 202.7; 2.45	IVMP, tacrolimus	3/3	No
22.M/41	Fever, cough, nausea, poor appetite	**Brain:** T2-hyperintense lesions and enhancement in meninges **Spine:** lesions in T10-S2	Antibody (-)	GFAP-IgG (1:100)	260; 664.1; 1.96	IVMP, ganciclovir	1/5	Yes
23.M/42	Limb weakness, consciousness disturbance	**Brain:** T2-hyperintense lesions and enhancement in bilateral basal ganglia, lateral ventricle and thalamus, corpus callosum, insular, temporal, occipital, and left hippocampus **Spine:** normal	NA	GFAP-IgG (1:32)	54; 755.4; 4.01	IVMP, ganciclovir, vidarabine	3/3	No
24.M/44	Fever, psychosis, headache, tics, limb weakness, consciousness disturbance, dysuria	**Brain:** T2-hyperintense lesions in bilateral basal ganglia, lateral ventricle, cerebellum, thalamus, hippocampus and cerebral peduncle, pons, mesencephalon, medulla oblongata Enhancement in pons and medulla oblongata **Spine:** lesions and enhancement in C1-C2	GFAP-IgG (1:10)	GFAP-IgG (1:100)	9; 273.0; 7.71	IVMP, IVIG, penciclovir	5/5	Yes
25.F/45	mouth numbness, forehead pain, intercostal pain	**Brain:** T2-hyperintense lesions in bilateral frontal, pons Enhancement in pons **Spine:** normal	NA	GFAP-IgG (1:32)	2; 290.4; 2.31	ganciclovir	1/1	No
26.F/45	blurred vision, limb numbness, facial pain, dysuria, constipation, fever	**Brain:** T2-hyperintense lesions in bilateral frontal, left parietal **Spine:** lesions and enhancement in C2-3 and T5	Antibody (-)	GFAP-IgG (1:32)	6; 401.9; 2.94	IVMP	3/2	Yes
27.M/47	Fever, dizziness, chest tightness, alalia, deviated mouth	**Brain:** normal **Spine:** NA	GFAP-IgG (1:32)	GFAP-IgG (1:100)	108; 954.5; 1.86	IVMP, IVIG, ganciclovir	3/6	Yes
28.M/48	Limb numbness, dysuria, constipation, limb pain	**Brain:** T2-hyperintense lesions in bilateral frontal **Spine:** lesions in C4-T1	Antibody (-)	GFAP-IgG (1:32)	2; 734.7; 4.04	IVMP, penciclovir	3/1	No
29.F/51	Fever, headache, nausea, vomiting, poor appetite, consciousness disturbance, limb-shaking, psychosis, abdominal distension	**Brain:** T2-hyperintense lesions in bilateral cerebral hemisphere, basal ganglia and thalamus, cerebellum, meninges Enhancement in meninges **Spine:** lesions in C4	GFAP-IgG (1:32)	GFAP-IgG (1:32)	108; 572.0; 2.94	IVMP, IVIG, RIT, acyclovir	3/3	Yes
30.F/55	Nausea, vomiting, facial pain, alalia, dysphagia, coughing when drinking water, limb numbness	**Brain:** T2-hyperintense lesions in bilateral frontal, lateral ventricle and parietal **Spine:** normal	AQP4-IgG (1:10)	GFAP-IgG (1:32) AQP4-IgG (1:32)	24; 368.1; 3.05	IVMP, ganciclovir	3/3	No
31.M/57	Hiccup, nausea, vomiting, chest tightness, belching, facial pain, limb weakness, limb numbness	**Brain:** T2-hyperintense lesions and enhancement in right basal ganglia and lateral ventricle, medulla oblongata **Spine:** normal	Antibody (-)	GFAP-IgG (1:32)	70; 452.6; 3.38	IVMP, ganciclovir	3/1	No
32.M/59	Fever, headache, limb-shaking, poor appetite, nausea, psychosis	**Brain:** T2-hyperintense lesions in bilateral basal ganglia, lateral ventricle, cerebellum and thalamus, left frontal **Spine:** NA	GFAP-IgG (1:32)	GFAP-IgG (1:100)	30; 759.4; 2.67	IVIG, penciclovir	1/0	No
33.F/68	Fever, limb weakness, limb numbness, alalia, limb-shaking	**Brain:** T2-hyperintense lesions in bilateral basal ganglia, lateral ventricle, cerebellum, frontal and parietal, pons Enhancement in bilateral basal ganglia, lateral ventricle and cerebellum, pons **Spine:** normal	NA	GFAP-IgG (1:32)	2; 570.8; 3.84	IVMP, penciclovir	4/4	No

### 3.1 Demographic data and clinical manifestations

The median age at disease onset was 28 years (range: 2–68 years), and 15 patients were children with a median age of 8 years (range: 2–14 years). There were 17 males and 16 females in the cohort. Of these, 16 cases were positive for GFAP-IgG in both the CSF and serum, while 17 cases were positive for GFAP-IgG only in the CSF. GFAP antibody titers in the serum and CSF were not significantly associated with the disease severity on admission or short-term prognosis (P = 0.17, r_s_ = 0.26; P = 0.60, r_s_ = -0.10; P = 0.20, r_s_ = -0.23; P = 0.15, r_s_ = -0.26; respectively).

**Figure 2:**
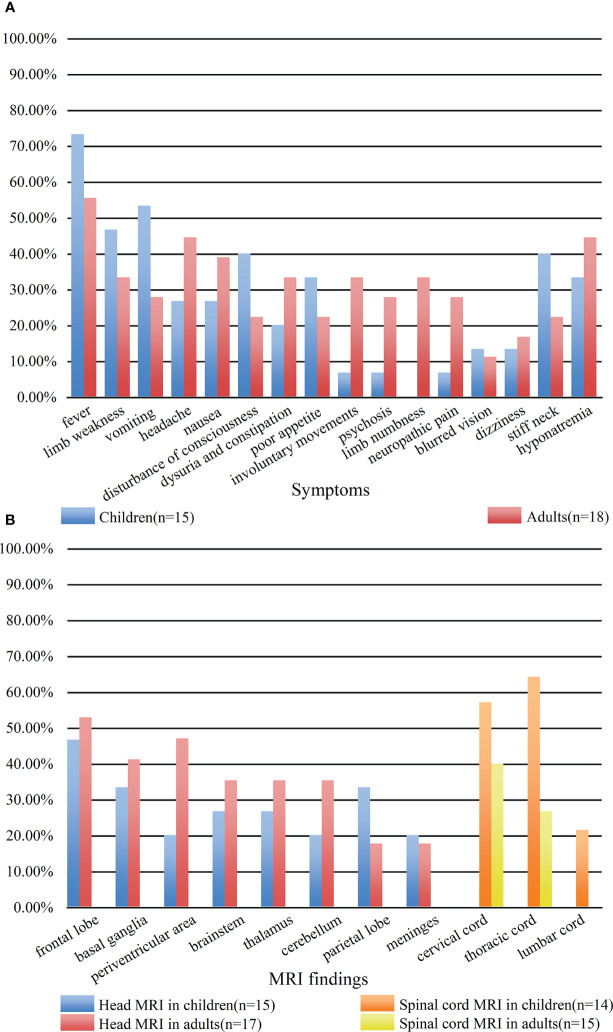
Comparison of clinical symptoms **(A)** and MRI findings **(B)** between children and adults. **(A)** The blue bar represented the clinical symptoms of children. The red bar represented the clinical symptoms of adults. **(B)** The blue bar represented the head MRI findings of children. The red bar represented the head MRI findings of adults. The orange bar represented the spinal MRI findings of children. The yellow bar represented the spinal MRI findings of adults.

A total of 29 patients had three or more symptoms at admission. However, there was no statistical correlation between the number of symptoms and disease severity on admission or short-term prognosis (P = 0.05, r_s_ = 0.34; P = 0.45, r_s_ = 0.14 respectively). Seventeen of the thirty-three patients had prodromal symptoms, including fever (n = 15), vomiting (n = 2). The main clinical symptoms were fever (21 cases); limb weakness (13 cases); vomiting (13 cases); headache (12 cases); nausea (11 cases); disturbance of consciousness (10 cases); dysuria and constipation (9 cases); poor appetite (9 cases); involuntary movements, including tics and limb-shaking (7 cases); psychosis (6 cases); limb numbness (6 cases); neuropathic pain involving the face, limbs, and intercostal region (6 cases). Thirteen patients had hyponatremia (<135 mmol/L); four patients presented with blurred vision; and three patients had focal epilepsy. Ten patients showed neck stiffness on physical examination. Patient #27 died of acute brainstem failure during hospitalization, and patient #29 died of severe pneumonia 3 months after discharge. **Figure 2A** compared the symptoms in children and adults. And only adult patients presented with limb numbness in our cohort.

### 3.2 Laboratory examination

Thirteen patients had abnormal tumor markers in the serum, mainly ferritin (5/13) and neuron-specific enolase (5/13). However, none of the patients were diagnosed with a tumor as of July 2022. Viral antibodies (immunoglobulin M) were detected in the serum of 19 patients, which primarily comprised antibodies to Epstein-Barr virus (12/19), cytomegalovirus (11/19), and coxsackie virus (7/19). Ten patients had non-neural autoantibodies in the serum, including anti-SSA antibody (6/10), antinuclear antibody (5/10), rheumatoid factor (1/10), and anti-PM-Scl antibody (1/10) (**Table 2**).

**Table 2:** Serological findings of 33 patients with autoimmune GFAP astrocytopathy.

Serological findings	Patients
**abnormal tumor markers**	**13/33**
Ferritin	5/13
neuron-specific enolase	5/13
tumor-associated antigen 72-4	3/13
carcinoembryonic antigen	2/13
tumor associated antigen 125	1/13
non-small cell lung cancer antigen 21-1	1/13
alpha-fetoprotein	1/13
tumor-associated antigen 19-9	1/13
**viral antibodies (immunoglobulin M) in serum**	**19/32**
Epstein-Barr virus	12/19
Cytomegalovirus	11/19
coxsackie virus	7/19
measles virus	6/19
herpes simplex virus I	3/19
human parvovirus B-19	2/19
influenza b virus	1/19
parainfluenza virus	1/19
Adenovirus	1/19
**non-neural autoantibodies in the serum**	**10/33**
anti-SSA antibody	6/10
antinuclear antibody	5/10
rheumatoid factor	1/10
anti-PM-Scl antibody	1/10

CSF abnormalities were found in 30 patients. Pleocytosis was found in 27 patients (mainly lymphocytes), with the highest number being 950 × 10^6^/L (reference range, 0–5 × 10^6^/L). There were 19 cases with elevated proteins up to 4433 mg/L (reference range, 150–450 mg/L). Furthermore, 10 patients showed hypoglycorrhachia, with the minimum value being 1 mmol/L (reference range, 2.5–4.5 mmol/L). Viruses were detected in the CSF of five patients, namely Epstein-Barr virus (3/5), enterovirus (1/5), and herpes simplex virus I (1/5). *Aspergillus fumigatus* was detected in the CSF of patient #12. Oligoclonal antibodies (IgG) were identified in the CSF of four patients (**Table 3**).

**Table 3:** CSF findings of 33 patients with autoimmune GFAP astrocytopathy.

CSF findings	Patients	Median (range)
**CSF abnormalities**	**30/33**	
pleocytosis (>5 × 10^6^/L)	27/33	38 (1-950)
Elevated proteins (>450.0 mg/L)	19/33	572.0 (202.7-4433.0)
hypoglycorrhachia (<2.50 mmol/L)	10/33	2.95 (1.00-7.71)
**viral antibodies (immunoglobulin M) in CSF**	**5/33**	
Epstein-Barr virus	3/5	
enterovirus	1/5	
herpes simplex virus I	1/5	
**Oligoclonal antibodies (IgG)**	4/33	

Of the 33 patients, eight patients had one or more overlapping neural antibodies in the CSF or serum, including seven females and one male. AQP-4 was the most common overlapping antibody in the CSF of GFAP-A patients, followed by MOG and NMDAR. Furthermore, all patients with overlapping AQP4 antibodies responded poorly to first-line immunotherapy (IVMP, IVIG) in the acute phase, and two of them presented with longitudinally extensive transverse myelitis. **Table 4** listed the clinical manifestation and MRI characteristics of patients with coexisting neural antibodies.

**Table 4:** Clinical manifestation and MRI characteristics of patients with coexisting neural antibodies.

Types of coexisting antibodies		Clinical manifestation	MRI characteristics	
			Head	Spinal cord
AQP4	Patient #13	vomiting, blurred vision, walk unsteadiness **(LETM)**	normal	medulla oblongata and C1-C5
	Patient #21	limb numbness, tics **(LETM)**	NA	medulla oblongata, C1-6 and T3-5
	Patient #30	nausea, vomiting, facial pain, alalia, dysphagia, coughing when drinking water, limb numbness **(encephalitis)**	frontal, lateral ventricle and parietal	normal
MOG	Patient #2	blurred vision **(ON)**	frontal, parietal, temporal, corpus callosum, pons, cerebellum, optic nerve	C4-T8
	Patient #16	Fever, headache, nausea, vomiting, dysuria, constipation, blurred vision, limb weakness, epilepsy **(encephalitis, myelitis)**	cerebellum and thalamus	normal
MOG and NMDAR	Patient #11	limb weakness, lethargy, poor appetite **(encephalitis, LETM)**	normal	C2-6 and T9-12
AQP4, NMDAR, GAD651, and GLYR1	Patient #18	headache, dizziness, lethargy, psychosis **(encephalitis)**	frontal and lateral ventricles enhancement in the optic chiasm, bilateral anterior portion of the thalamus, fornix column and triventricular area	normal
YO	Patient #20	dizziness, limb weakness, poor appetite **(encephalitis)**	frontal	NA

LETM, longitudinally extensive transverse myelitis; ON, optic neuritis.

### 3.3 MRI findings

**Figure 3:**
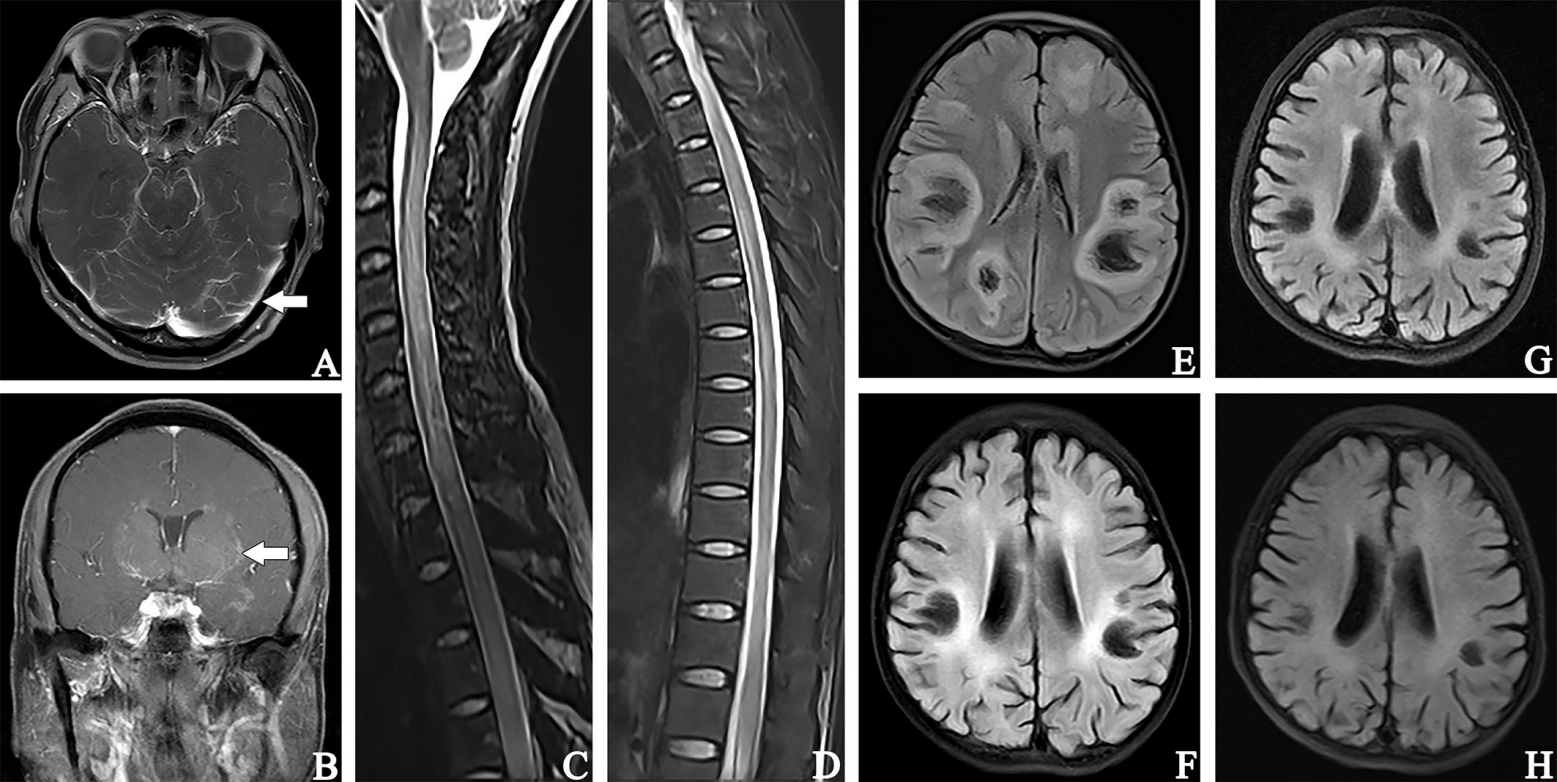
Contrast-enhanced T1-weighted magnetic resonance image **(A, B)**. **(A)** patient #18, showed soft meningeal linear enhancement (arrow). **(B)** patient #23, MR image suggested patchy enhancement around the lateral ventricles (arrow). **(C, D)**: patient #18, T2-hyperintense lesions in C2-T12. Fluid attenuated inversion recovery **(E–H)**. MR images of patient #3. **(E)** MR image showed multiple lesions of bilateral brain parenchyma at admission. **(F)** (1 month after treatment with IVMP and IVIG) and **(G)** (2 months after treatment) showed that the extent of lesions gradually decreased and brain atrophy began to appear. **(H)** 4 months after treatment, a significant reduction in the lesion range and high-signal lesions in the bilateral lateral periventricular. The widening and deepening of the cerebral sulcus and fissure were evident.

All 33 cases underwent MRI examinations, including 32 head MRIs and 29 spinal cord MRIs. MRI abnormalities were mainly T2 sequence high signal lesions, which appeared as patchy, linear, punctate, and stripe patterns. Results from head MRIs were varied: four patients had normal imaging, 28 patients demonstrated abnormal T2 hyperintensities, and 12 of 20 patients showed abnormal contrast enhancement. The lesions were mainly located in the frontal lobe (16/32), basal ganglia (12/32), periventricular area (11/32), brainstem (10/32), cerebellum (9/32), thalamus (9/32), parietal lobe (8/32), and meninges (6/32); enhancement patterns were observed in the meninges (n = 6), periventricular area (n = 3), and basal ganglia (n = 3) (**Figures 3A, B**). Regarding spinal cord MRI, T2 hyperintensities were observed in the cervical cord (14 cases), followed by the thoracic cord (13 cases). Five of nine patients who underwent contrast-enhanced spinal MRI showed abnormal enhancement. The lesion of patient #17 involved up to 18 spinal cord segments (**Figures 3C, D**). Additionally, there was no statistical difference in abnormal spine cord MRIs between children and adults (P = 0.264). **Figure 2B** compared the MRI characteristics between adults and children. Besides, the MRI results revealed that the lesions in GFAP-A patients generally reduced in size or disappeared after immunotherapy. Patient #3 presented with multiple lesions in the brain parenchyma on admission MRI (**Figure 3E**). Re-examination of MRI at 1 and 2 months after treatment with IVMP and IVIG showed that the lesions gradually reduced (**Figures 3F, G**). Four months after the treatment, his MRI lesions were significantly reduced in size (**Figure 3H**). Moreover, his symptoms were fully resolved 6 months after the onset.

### 3.4 Treatment, outcome, and prognosis analysis

**Figure 4:**
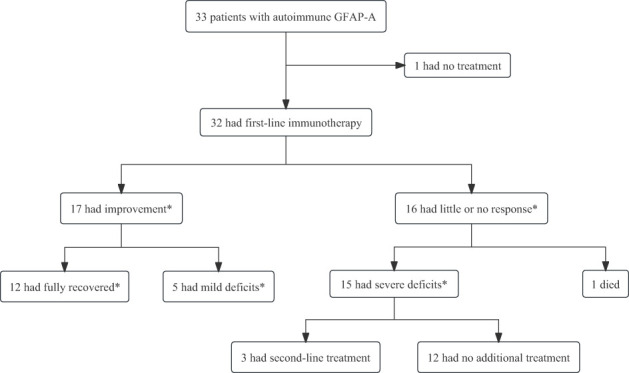
The treatment response and short-term outcomes of patients with autoimmune GFAP astrocytopathy. First-line immunotherapy included intravenous methylprednisolone, intravenous immunoglobulin, and plasma exchange, alone or in combination. Second-line immunotherapy included rituximab, tacrolimus, or mycophenolate mofetil. *The occurrence of improvement or absence of improvement was assessed at 4 weeks after the initiation of immunotherapy. Recovered was defined as mRS=0; mild deficits were defined as mRS= 1,2; and severe deficits were defined as mRS= 3,4.

Treatment responses and short-term outcomes are summarized in **Figure 4**. Most patients experienced a significant improvement in their symptoms after first-line immunotherapy (P = 0.02). Among the 33 patients, 17 were treated with IVMP only; 14 underwent IVMP plus IVIG therapy; 1 patient had only IVIG treatment. And 1 patient received PE for poor outcome after receiving IVMP plus IVIG. Nineteen patients underwent oral tapering of steroids in the maintenance period. Three patients responded poorly to treatment and subsequently received immunosuppressive therapy (MMF, RIT, and TAC). Moreover, five patients were mechanically ventilated because of respiratory failure. 19 proven viral infection and 7 suspected viral infection were treated with antiviral drugs in the early stage of admission.

The median mRS score at admission was 3 and that at 4 weeks after the initiation of immunotherapy was 2. Short-term prognosis was significantly better in children than in adults (P = 0.04). In the univariate binary logistic model, the factors associated with poor outcomes included positive non-neural autoantibodies and proven viral infection (P = 0.03, 0.03, respectively). Multivariate binary logistic regression model identified positive non-neural autoantibodies and proven viral infection as the independent factors associated with a poor outcome (P = 0.03, 0.02, respectively) (**Table 5**).

**Table 5:** Factors associated with poor outcomes at 4 weeks after the initiation of immunotherapy (mRS ≥3).

	Odds ratio (95% CI)	P
Univariable analysis		
Demographic data		
age of onset	1.03 (0.99-1.07)	0.11
gender	0.69 (0.18-2.73)	0.60
hospital days	1.01 (0.97-1.05)	0.58
Serologic data		
MLR*	1.66 (0.22-12.54)	0.63
NLR*	0.98 (0.91-1.06)	0.66
tumor marker	0.86 (0.21-3.47)	0.83
proven viral infection in serum	5.71 (1.16-28.07)	**0.03**
positive for non-neural autoantibodies in serum	7.50 (1.28-44.09)	**0.03**
CSF examination		
white cells	1.002 (0.997-1.007)	0.45
protein level	1.000 (0.999-1.001)	0.66
viral antibodies in CSF	5.33 (0.53-54.03)	0.16
glucose level	1.89 (0.78-4.60)	0.16
Clinical symptoms		
fever	0.91 (0.22-3.76)	0.90
hyponatremia	1.43 (0.35-5.79)	0.62
headache	0.65 (0.16-2.72)	0.56
disturbance of consciousness	3.63 (0.74-17.81)	0.11
nausea	0.83 (0.20-3.56)	0.81
vomiting	1.43 (0.35-5.79)	0.62
limb weakness	0.86 (0.21-3.47)	0.83
dizziness	5.33 (0.53-54.03)	0.16
stiff neck	1.09 (0.25-4.82)	0.91
limb numbness	1.08 (0.18-6.32)	0.94
dysuria and constipation	0.42 (0.09-2.01)	0.29
involuntary movements	3.41 (0.56-20.94)	0.19
neuropathic pain	1.08 (0.18-6.32)	0.94
psychosis	2.50 (0.39-16.05)	0.33
poor appetite	1.48 (0.32-6.90)	0.62
blurred vision	0.31 (0.03-3.35)	0.34
prodromal symptoms	0.89 (0.23-3.49)	0.87
ICU admission	1.87 (0.44-7.85)	0.39
mechanical ventilation	5.33 (0.53-54.03)	0.16
mRs at admission	1.44 (0.81-2.57)	0.21
Multivariable analysis		
positive for non-neural autoantibodies in serum	17.67 (1.60-195.11)	0.02
proven viral infection in serum	11.96 (1.26-113.92)	0.03

*MLR, monocyte-to-lymphocyte ratio; NLR, neutrophil-to-lymphocyte ratio.

At the last follow-up, seven patients had poor outcomes and 26 patients had good outcomes, with the median mRS score being 0. The average follow-up duration was 12 months, ranging from 3 to 47 months. 8 of 33 patients were hospitalized more than two times, including two patients who were hospitalized seven times (Patients #13 and #21). In addition, four patients relapsed during oral tapering of steroids: two of them had two relapses; one patient had three relapses; and one patient had five relapses. Three patients experienced worsening or recurrence of previous symptoms, and one patient had new symptoms which were significantly alleviated after immunotherapy.

## 4 Discussion

GFAP-A is a relatively rare autoimmune inflammatory CNS disorder. Despite several studies (2, 3, 7–13), there is limited understanding of its short-term prognosis, which motivated us to conduct this study. Our findings expanded the spectrum of symptoms, which comprise nausea, vomiting, poor appetite, and neuropathic pain. Importantly, patients with positive non-neural autoantibodies and proven viral infections in the serum at admission were found to have a poor short-term prognosis, which was rarely reported before. Additionally, our study results differ from those of previous studies in that adult patients were more likely to have sensory symptoms such as limb numbness and poor short-term prognosis. Hence, the present study provides a relatively comprehensive description of the clinical characteristics and short-term prognosis of GFAP-A.

The median age of disease onset in GFAP-A patients was 28 years (range: 2–68 years) in our study, while it was 40–50 years (range: 11 months to 103 years) in previous studies (8, 10). We think that the difference may be because eight patients in our study were enrolled from children’s hospitals. Our study confirmed that 29 patients with autoimmune GFAP-A had three or more clinical symptoms, which were diverse and non-specific. We also found other clinical presentations, including nausea and vomiting, which were rarely mentioned previously. The underlying mechanisms were heterogeneous, and included area postrema syndrome, hyponatremia, meningitis, and encephalitis. Poor appetite was not a rare symptom in our study, owing to GFAP expression by the enteric glial cells. It is an important component of the enteric nervous system, which regulates enteric neural reflexes and maintains intestinal homeostasis (14, 15). We also found that limb numbness was more likely to occur in adults, which suggests that adults are more prone to sensory disturbance in autoimmune GFAP-A. In contrast to the findings of Zhuang et al. (8), myelitis in children was also relatively common in our cohort, which was supported by no statistical difference in abnormal spinal cord MRIs between children and adults. As already noted by Flanagan et al., most patients had infectious prodromal symptoms, which indicates that autoimmune GFAP-A may be triggered by infection (2, 9). Additionally, hiccups were the main symptom of area postrema syndrome in autoimmune GFAP-A, supported by MRI findings of T2-hyperintense lesions in the dorsal medulla oblongata (16). Interestingly, frequent neuropathic pain was reported in six cases, and patient #13 presented with left-sided peripheral facial nerve palsy at the first relapse. It is worth noting that GFAP is also expressed by Schwann cells and satellite glial cells of peripheral nerves (17).

Thirteen patients with GFAP-A had serum tumor markers detected in our study. Therefore, clinicians should give high priority to tumor screening in autoimmune GFAP-A patients, especially within 2 years of the onset (4). Additionally, we found that viral antibodies are frequently detected in the serum of autoimmune GFAP-A patients. More importantly, proven viral infection in the serum on admission suggests a poor short-term prognosis. Of note, viral infections are related to CNS autoimmune disorders (18). Although a close relationship between GFAP-A and viral infection has been previously demonstrated, all previous studies detected the virus in the CSF (11, 19–21). We speculate that serum viral antibodies enter the CNS through the blood-brain barrier and then participate in the pathophysiological process of autoimmune GFAP-A. The underlying mechanism still needs to be confirmed by further animal experiments. The other factor associated with poor short-term prognosis was overlapping non-neural autoantibodies, which were also encountered in 75% of the first Chinese cohort (11). Even though these antibodies are not specific per se, it suggests that clinicians should be alert for co-morbid autoimmune diseases other than CNS involvement in patients with GFAP-A, which may exacerbate the patient’s condition. Iorio et al. (13) also reported that GFAP-A in combination with other autoimmune diseases was common. In our study, we noted that eight patients had coexisting neural autoantibodies, with the most common being AQP-4 antibodies, which tend to occur in women. Similar to the findings of Xiao et al. (10), patients with overlapping AQP-4 antibodies responded poorly to immunotherapy in the acute phase, but the exact mechanism is unclear.

Our results provide statistical evidence that first-line immunotherapy is effective in most patients with autoimmune GFAP-A in the acute phase, which is more convincing than the results of previous observational studies. As recently described (9), relapse may occur during oral tapering of steroids in most patients. The recurrence rate was 12.1% in our cohort, which was lower than that reported in previous studies (18%) (7, 12). Relapses usually involved the worsening of previous symptoms, although a few patients developed new symptoms. In contrast to the findings of Xiao et al. (10), we noticed that one patient treated with TAC still had frequent relapses during follow-up, questioning the effectiveness of TAC for preventing a relapse. Intriguingly, we found children had a better short-term prognosis, which was reported to be poor previously.

Our study findings provide novel insights into the clinical characteristics and short-term prognosis of GFAP-A patients. However, there are some limitations of our study. Firstly, this was not a randomized and prospective study, but a precursor to future trials to explore the prognostic factors. Secondly, we only evaluated 33 patients including 15 children, which is a small sample size and the presence of population heterogeneity. In the future, more studies are needed in a larger population. Lastly, patients with Alzheimer’s disease and cancer have been shown to have serum GFAP antibodies (22). Therefore, the specificity of serum GFAP antibodies remains uncertain. We only included patients with positive CSF GFAP antibodies, which may have excluded some patients.

In conclusion, our study not only expands the known spectrum of clinical characteristics of GFAP-A, but also statistically confirms the effectiveness of first-line immunotherapy in the acute phase. Furthermore, we identified the prognostic factors associated with the short-term outcomes of GFAP-A as well as significant differences between children and adults with GFAP-A.

## Data availability statement

The raw data supporting the conclusions of this article will be made available by the authors, without undue reservation.

## Ethics statement

Written informed consent was obtained from the individual(s), and minor(s)’ legal guardian/next of kin, for the publication of any potentially identifiable images or data included in this article.

## Author contributions

NX and WZ contributed to conception and design of the study. YX and YW organized the database. FL performed the statistical analysis. WZ wrote the first draft of the manuscript. LW, YL, HL, and CW wrote sections of the manuscript. All authors contributed to manuscript revision, read, and approved the submitted version.
